# Supplementary material for: Single Cell Transcriptomic and Chromatin Profiles Suggest Layer Vb Is the Only Layer With Shared Excitatory Cell Types in the Medial and Lateral Entorhinal Cortex
Source: Front Neural Circuits. 2022 Jan 26;15:806154. doi: 10.3389/fncir.2021.806154 (PMC8826650; doi:10.3389/fncir.2021.806154)
Supplement: Supplementary file 14 [file Data_Sheet_1.ZIP › Exc_vs_Inh_Motifs/homerResults/motif14.similar.html]

motif14

## Information for motif14

T
A
G
C
G
A
T
C
G
C
A
T
A
T
G
C
T
C
A
G
G
C
T
A
C
G
T
A
A
G
T
C
G
A
C
T
T
G
A
C
  
Reverse Opposite:  

A
C
T
G
C
T
G
A
T
C
A
G
G
C
A
T
C
G
A
T
A
G
T
C
T
A
C
G
C
G
T
A
C
T
A
G
A
T
C
G
  

|  |  |
| --- | --- |
| p-value: | 1e-174 |
| log p-value: | -4.023e+02 |
| Information Content per bp: | 1.693 |
| Number of Target Sequences with motif | 3700.0 |
| Percentage of Target Sequences with motif | 8.77% |
| Number of Background Sequences with motif | 587.6 |
| Percentage of Background Sequences with motif | 5.41% |
| Average Position of motif in Targets | 376.5 +/- 258.0bp |
| Average Position of motif in Background | 360.2 +/- 258.1bp |
| Strand Bias (log2 ratio + to - strand density) | -0.1 |
| Multiplicity (# of sites on avg that occur together) | 1.15 |
| Motif File: | file (matrix) reverse opposite |

### Similar de novo motifs found

|  |  |  |  |  |  |  |  |
| --- | --- | --- | --- | --- | --- | --- | --- |
| Rank | Match Score | Redundant Motif | P-value | log P-value | % of Targets | % of Background | Motif file |
| 1 | 0.924 | A C T G A G T C A G T C A C G T A T C G T C A G C G T A C T G A A G T C A C G T A G T C G C T A | 1e-143 | -331.396641 | 3.12% | 1.42% | motif file (matrix) |
